# On the Use of Opium in Certain Forms of Nervous Irritability and Coma, Which Frequently Attend Typhus Fevers

**Published:** 1852-02

**Authors:** Jared Kirtland


*On the Use of Opium in certain forms of Nervous Irritability and Coma, which frequently attend Typhus Fevers.*—By Jared Kirtland, M. D. The beneficial effects of opium and its various combinations, in allaying irritation in our common forms of fevers, are well understood; but their peculiar adaptation to supporting the system and controlling the action of the nervous centres in the severe forms of Typhus Mitior of Cullen, have not, in all instances, been duly appreciated.

Under the present epidemic constitution, the typhoid quality of diseased action is comparatively rare, yet within the last half century it has prevailed extensively; and, at certain periods, in certain localities, has either constituted the great amount of disease, or imparted its peculiar characters to other species of diseased action.

From the year 1805 to 1830, all the diseases of the New England States were essentially typhoid in their quality; and from 1824 to 1831 such was the quality of disease in the north-eastern part of Ohio. Since then, with a few exceptions, our epidemic constitution has developed higher grades and qualities of disease; yet, we have no assurance that other changes may not again bring physicians in contact with disorders of a typhoid quality.

Diseased action has been governed in its changes at different periods by more uniform and definitive laws, than is usually supposed.

By *Typhoid*, I mean an asthenic form of fever, in which a deficiency of vitality in the system renders it incapable of acting with its natural and full force. I have no reference to the views of the modern French school. There is but one species of Asthenic continued Fever. TYPHUS, which, when it locates on the nervous centres, constitutes TYPHUS GRAVIOR, of Cullen, or the PUTRID FEVER, of Huxham.

And when it locates upon the *mucous tissues* of the alimentary canal, as it has been very wont to do, since the cholera first began to extend over Europe and this country, it gives rise to the pathological condition, known in some modern works as TYPHOID Fever, *dothinenteritis.*

This is not, however, a distinct species but a mere variety, arising from the nature and character of the part on which it locates.

When the nervous centres become the primary point of attack, irritated action ensues, giving origin to peculiar symptoms which characterize Nervous Fever, of common language.

This irritated action is a very different morbid condition from inflammation, and though it is attended by an action of the brain, it is intimately connected with, and dependent on a reduced amount of the principle of life—vitality.

If left to take its own way in low forms of Nervous Fever, it is liable to result in a great diminution of thepowers of the brain, and as a consequence, a state of torpor will ensue, manifested by Coma.

In the nervous type of Typhus Fever we therefore expect to find either nervous irritability or Coma—conditions very nearly allied in their pathology, one producing the other.

Opium, or its combinations may be successfully used as a remedy in both.

In the former it should be employed in uniform doses, sufficiently heavy to control the morbid action, and repeated so frequently as to support the system. Other means may also be required to aid in accomplishing the purpose.

In Typhoid Coma, the life of the patient must be, in a great measure, staked upon opium.

To proceed prudently we must make out clearly a correct diagnosis; we must satisfy ourselves that the deep sleep, stertorous breathing, and loss of consciousness, proceed from exhaustion of the energy of the nervous centres, and not from sympathy with a loaded prima via, or fullness of the vessels of the brain. The indication then becomes clear, that the small stock of vitality must be economized and the safest means pursued to reaccumulate it. For this purpose opium is the main agent.—As a general rule it must be used, for adults about one grain, or its equivalent of morphine, every two hours, in connection with suitable adjuvants.

The first effect will be to arouse the patient in a slight degree from his slumbers. If the dose be suitably repeated so as to keep up the stimulus, perfect wakefulness and consciousness will ultimately be established, and the condition will gradually improve from day to day. If, however, the physician or the attendants view the attempt to create a state of wakefulness from heavy coma, by this article, as an absurdity, the patient will probably be lost. At this juncture scepticism is fatal; and instability of purpose may defeat the best design.

I am aware, that I have advanced a doctrine novel to many, and perhaps wild, visionary and dangerous in the estimation of all.

In regard to its novelty, I will observe that it claims the merit of age. Cullen, in his Materia Medica, under the article OPIUM, gives us to understand that in one kind of Phrenitis, stimulants and narcotics are dangerous and injurious, but that there is *one kind of inflammation* of the brain in which opium is the main remedy.

The kind of inflammation to which he doubtless referred is this irritated and exhausted condition of the nervous centres.—*Minutes of the Ohio State Medical Society.*

